# The Danger Zone in the Anterior Neck: Anatomical Landmarks to Avoid Injury to Anterior Jugular Vein During Face-Lift and Neck-Lift

**Published:** 2018-01-19

**Authors:** Andrew M. Swiergosz, J. Stephen Gunn, Steven A. Schulz, Joshua T. Henderson, Joshua H. Choo, Arian Mowlavi, Bradon J. Wilhelmi

**Affiliations:** University of Louisville School of Medicine, Louisville, Ky

**Keywords:** anterior jugular vein, anterior neck, face-lift, neck-lift, rhytidectomy

## Abstract

**Background**: An estimated 125,711 face-lifts and 54,281 neck-lifts were performed in 2015. Regardless of the technique employed, facial and neck flap elevation carries with it anatomical risk of which any surgeon performing these procedures should be aware of. Statistics related to anterior jugular vein injury during these procedures have not been published. **Objective**: To define a “danger zone” that will contain both of the anterior jugular veins on the basis of anatomical landmarks to aid surgeons with planning their surgical approach during rhytidectomy in the anterior neck region. **Methods**: Ten fresh tissue heminecks were dissected. All specimens were dissected under loupe magnification in a 45° (face-lift) position in which a midline incision was used for exposure. Measurements from the anterior jugular vein to the hyoid, thyroid cartilage, and cricoid cartilage bilaterally were taken. The transverse distance between the anterior jugular veins at the level of the hyoid, thyroid cartilage, and cricoid cartilage was also measured. **Results**: The anterior jugular veins remain in an anatomical danger zone while they travel in the anterior neck. Regardless of anatomical variation of the vessels between bodies, they generally reside in this danger zone from their inferior emergence behind the sternocleidomastoid muscle until they branch in the suprahyoid region. **Conclusions**: Knowledge of the anatomy, course, and location of the anterior jugular veins through the anterior neck based on anatomical landmarks and distance ratios can facilitate a safer dissection during rhytidectomy procedures.

Rhytidectomy is one of the most commonly performed cosmetic surgical procedures in the United States. In 2015, there were more than 125,000 surgical procedures performed.^1^ With the prevalence of this procedure being so high, it is of utmost importance that the surgeon be aware of measures available to avoid and prevent complications. The most common complication after a rhytidectomy is hematoma, with incidence ranging from 2% to 9%.^2^ These hematomas can lead to additional problems including scarring of subcutaneous tissue, delay in wound healing, tissue ischemia, and skin necrosis.^2^ Avoiding the unnecessary injury of large vessels during rhytidectomy is imperative to minimizing these complications. One vessel in the neck that is particularly vulnerable to injury during rhytidectomy is the anterior jugular vein. Many surgeons are aware of the external jugular veins and how to avoid them, but there is less literature about the anterior jugular veins and techniques to predict their location and avoid them.

## METHODS

**Figure 1:**
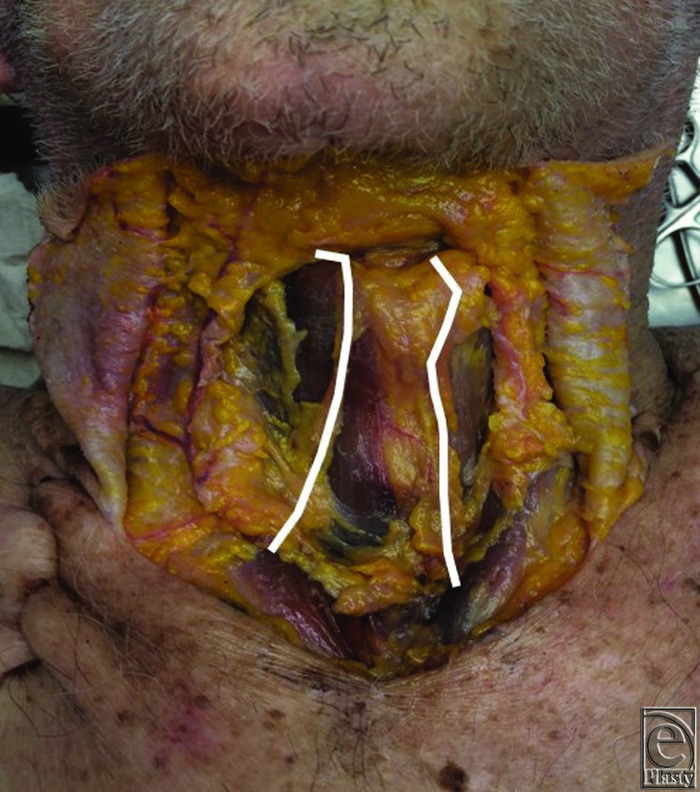
A cadaveric specimen demonstrating the location and course of the anterior jugular veins in the anterior neck.

Dissection was carried out under loupe magnification on 10 Caucasian fresh heminecks to determine the location of the anterior jugular veins as they course through the anterior neck (Fig 1). The sample group consisted of 4 males and 6 females. Surgical exposure was initiated with a superficial midline incision from the sternal notch to the mental protuberance. Another superficial incision was made along the superior margin of the clavicles bilaterally beginning at the original midline incision and extending laterally to the midpoint of the clavicle. A final superficial incision was made along the inferior border of the mandible from the midline medially to the angle of the mandible laterally. Sharp dissection was used to elevate skin flaps bilaterally and fully expose the sternocleidomastoid muscle (SCM). Care was taken to avoid injury of the anterior jugular veins as they emerged along the anterior border of the SCM. The transverse distance was measured from the anterior jugular veins to the midline of the hyoid, the midline of the thyroid cartilage, and the midline of the cricoid cartilage. In addition, the distance between the 2 anterior jugular veins was measured at the level of the hyoid, the thyroid cartilage, and the cricoid cartilage. The distance was also measured from the sternal notch to the point at which the anterior jugular vein emerges from behind the SCM as well as the distance of the most superior portion of the anterior jugular vein to the menton. Statistical data were calculated as the average measurement ± standard deviation.

## RESULTS

**Figure 2:**
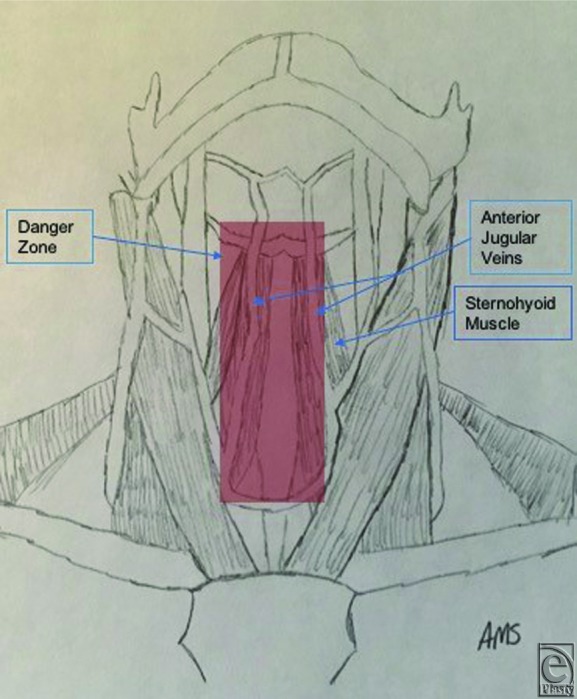
Suggested danger zone of the anterior neck that contains the anterior jugular veins.

In the 10 dissections, the specimens had an average neck circumference of 42.8 cm and an average neck height, measured from the sternal notch to the menton, of 13.9 cm. The right anterior jugular vein was found to be an average of 0.98 cm ± 0.39 cm from the midline of the hyoid, 1.02 cm ± 0.36 cm from the midline of the thyroid cartilage, and 0.98 cm ± 0.47 cm from the midline of the cricoid cartilage. The left anterior jugular vein was found to be an average of 1.11 cm ± 0.56 cm from the midline of the hyoid, 0.68 cm ± 0.27 cm from the midline of the thyroid cartilage, and 0.38 cm ± 0.17 cm from the midline of the cricoid cartilage. The average distance between the anterior jugular veins was found to be 1.81 cm ± 0.62 cm at the level of the hyoid, 1.64 cm ± 0.28 cm at the level of the thyroid cartilage, and 1.4 cm ± 0.52 cm at the level of the cricoid cartilage. The average distance from the manubrium that the anterior jugular vein emerges from behind SCM on the right and the left was found to be 2.18 cm ± 0.85 cm and 3.06 cm ± 0.74 cm, respectively. As a result of these measurements, we propose an 8 cm tall x 1.5 cm wide danger zone overlying the sternohyoid (Fig 2).

## DISCUSSION

One of the most frequently encountered complications after rhytidectomy is hematoma.^3^ Hematoma can lead to additional issues complicating the patient's recovery. Hematomas may differ in severity from minor bruising requiring only conservative management to large expanding collections in need of emergent surgical drainage.^4^ An expanding hematoma may endanger the vascularity of skin flaps. In the short term, this can lead to edema, bruising, or seroma. The long-term consequence may even lead to regional hyperpigmentation and contour changes due to subcutaneous scarring.^3^ Additional potential sequelae include an increased risk of infection, need for additional intervention, risk for delayed wound healing, and skin flap necrosis.^4^ While techniques such as drain placement, compression dressings, and the use of tissue sealants^4^ have been used in an attempt to avoid postoperative hematoma, careful surgical technique, taking care to avoid the unnecessary damage of blood vessels in the area, reduces the risk of bleeding and hematoma formation.

The anterior jugular veins usually originate in the suprahyoid region through the union of several superficial veins. They descend between the midline and the anterior border of the SCM bilaterally.^5^ Near the thoracic level of the neck, the anterior jugular veins pass beneath the SCM and join with the subclavian veins or the external jugular veins.^5^

There are multiple methods to perform a rhytidectomy. Some of these approaches are more likely to injure the anterior jugular vein than others. The composite rhytidectomy dissection as described by Hamra^6^ consists of repositioning a composite musculocutaneous flap containing the platysma muscle, cheek fat, and the orbicularis oculi muscle. During this procedure, the creation of the neck flap carries the risk of injuring the anterior jugular vein. The lateral cervical dissections are done in a preplatysmal plane, leaving all fat on the flap, and an incision is made in the submental crease, joining the left and right cervical dissections.^6^ The manipulation of the platysma during this portion of the procedure can result in injury to the anterior jugular veins if appropriate precautions are not taken.

The corset platysmaplasty is another rhytidectomy technique that poses a risk of injury to the anterior jugular veins. As part of this procedure, a submental incision is made following the arch of the submental jawline.^7^ Through this incision, the central skin of the neck is undermined, elevating the anterior neck flap down to a level 2 to 3 fingerbreadths above the suprasternal notch.^7^ Any remaining subcutaneous fat is then removed from the medial platysma area. The platysma is then plicated in the midline beginning at the chin and continued downward to at least the level of the cricoid cartilage.^7^ Working in the anterior neck during this portion of the procedure can result in injury to the anterior jugular veins.

Other methods for rhytidectomy, such as the lateral overlapping plication of the platysma, as described by Gonzalez,^8^ do not pose a substantial threat to the anterior jugular veins. In this procedure, a vertical incision is made on the platysma beginning 1 cm below the mandibular border and running in the sense of its fibers. This creates anterior and posterior edges of muscle, allowing access to the subplatysmal plane.^8^ The overlapping is done by pulling the anterior edge of the muscle backward and cephalic.^8^ By laterally placating the platysma and avoiding working in the anterior neck, the anterior jugular veins remain safe and are unlikely to be injured.

The anatomy of the anterior jugular veins as it related to rhytidectomy has not previously been investigated in depth. The injury potential of these vessels in the anterior neck during rhytidectomy is a concern, as it adds to the bleeding risk and could be another source for hematoma formation.

## CONCLUSION

The anterior jugular veins are found in a danger zone defined by an 8 × 1.5-cm zone overlying the sternohyoid muscles. With these anatomical landmarks in mind, the surgeon can accurately predict the location of the anterior jugular veins as they course through the anterior neck and reliably proceed with flap dissection using caution inferiorly to the hyoid bone in the midline during rhytidectomy procedures.
